# Inflammatory and Noninflammatory Itch: Implications in Pathophysiology-Directed Treatments

**DOI:** 10.3390/ijms18071485

**Published:** 2017-07-10

**Authors:** Lai-San Wong, Tiffany Wu, Chih-Hung Lee

**Affiliations:** 1 Department of Dermatology, College of Medicine, Chang Gung Memorial Hospital and Chang Gung University, Kaohsiung 833, Taiwan; a9252@cgmh.org.tw; 2 Zanvyl Kreiger School of Arts and Sciences, Johns Hopkins University, Baltimore, MD 21287, USA; twu33@jhu.edu

**Keywords:** itch, atopic dermatitis, pruritogens

## Abstract

Itch is the main chief complaint in patients visiting dermatologic clinics and has the ability to deeply impair life quality. Itch results from activation of cutaneous nerve endings by noxious stimuli such as inflammatory mediators, neurotransmitters and neuropeptides, causing itch signal transduction from peripheral skin, through the spinal cord and thalamus, to the brain cortex. Primarily noninflammatory diseases, such as uremic pruritus, cause itch through certain pruritogens in the skin. In inflammatory skin diseases, atopic dermatitis (AD) is the prototypic disease causing intensive itch by aberrant skin inflammation and epidermal barrier disruption. Recent understanding of disease susceptibility, severity markers, and mechanisms have helped to develop targeted therapy for itch in AD, including monoclonal antibodies against IL-4, IL-13, thymic stromal lymphopoietin (TSLP), IgE and IL-31. Promising effects have been observed in some of them. In this review, we summarized targeted therapies for inflammatory itch in AD and for managing abnormal itch transductions in other common itching skin diseases.

## 1. Introduction

Itch is an unpleasant sensation that elicits the desire to scratch in order to remove noxious stimuli [1]. Itch is the most common chief complaint in patients visiting dermatology clinics and is analogous to cough and sneeze of the lower and upper respiratory tract, all three of which are host actions trying to clear noxious stimuli. Importantly, albeit the common presentation of these symptoms, they are occasionally difficult to treat. The pathomechanisms of these symptoms remain largely to be determined; thus, from the clinical and physiological aspects, this summary is aimed to focus on the pathophysiology of itch.

Despite great strides in the medical community, knowledge of how the brain senses and perceives itch is limited. Itch perception results from the activation of small unmyelinated skin nerve endings by pruritogens in skin to the ipsilateral dorsal root ganglion of the spinal cord. The signal is then relayed from the synapse to the contralateral spinothalamic tract then thalamus, and radiated to the neurons located in the sensory brain cortex [2]. Anything interfering in this pathway could result in abnormal itch perception. Therefore, potential treatments of itch could target any of the components in this pathway. The mechanisms in this pathway are complex and can be categorized into peripheral and central aspects. The peripheral aspect likely involves inflammatory mediators and neuromediators from skin and peripheral nerves, while the central aspect likely involves various neurotransmitters and neuropeptides from the central nerve system (CNS).

For peripheral sensory input, sensory C nerve fibers are equipped with a large number of different sensory receptors. The activation of pruritus-specific neurons by classical pruritogens, such as the histamine H1 receptor and proteinase-activated receptors (PAR-2 and PAR-4), could induce distinct types of pruritus. The inflammatory and soluble mediators derived from skin immune cells, such as dendritic cells, mast cells, and eosinophils, may activate the peripheral nerves, which may then release further pruritogens and neuropeptides. The neuropeptides released may form an itch circuit by influencing macrophages, T cells, dendritic cells, and eosinophils in the skin. The components of neuropeptides, soluble mediators, and cell types involved in the peripheral circuit are discussed in the following section.

In the CNS, several molecules attribute to itch perception and propagation, including gastrin-releasing peptides (GRP), opioid receptors, neuropeptides, and neurotransmitters. GRP and its receptor are reported to be involved in the central processing of itch [3]. The μ-opioid receptor (MOR) isoform MOR1D heterodimerizes with gastrin-releasing peptide receptor (GRPR) in the spinal cord and relays itch signals [4]. A recent study showed that mice deficient in natriuretic polypeptide b (Nppb) in dorsal root ganglion display a selectively impaired scratching response to a variety of pruritogens [5]. Note, however, that there remain debates about the role of Nppb and its interactions with GRP in the CNS [6]. On the level of the spinal cord, mice lacking Bhlhb5+ (B5-I) interneurons in the dorsal horn of the spinal cord exhibit enhanced scratching and skin lesions [7]. Recent studies identified a group of itch-specific neurons characterized by Mas-related G protein-coupled receptors A3 (MrgprA3) which are activated by chloroquine but not histamine [8]. Subsequently, an itch-specific population of MrgprA3-expressing neurons in the dorsal root ganglion was identified, along with multiple synapses with nearby neurons expressing gastrin-releasing peptide receptors (GRPRs, see below). Specific ablation of these neurons diminished the pruritogen-induced scratching responses but not pain responses. Transient receptor potential (TRP) activation in these cells releases dynorphin, a ligand for κ-opioid receptor, to alleviate itch [9]. In this review, we summarize the current knowledge about the pathophysiological mechanisms of inflammatory and noninflammatory itch. Based on the mechanisms, the clinical effectiveness and significances of the potential targeting treatments on itch are updated.

## 2. Itch in Primarily Noninflammatory Diseases

### 2.1. Psychogenic Pruritus

Psychogenic pruritus is considered psychiatric in origin and presents as itch in conditions such as obsessive-compulsive disorders, depression, and delusions of parasitosis [10,11]. It is characterized as an excessive impulse to scratch or pick at normal skin. The self-inflicting and gratuitous actions often result in bizarre-looking and repeated skin ulcerations and erosions. Treatments may include antipsychotic drugs, supportive measures, and psychotherapies.

### 2.2. Uremic Pruritus

Pruritus and dry skin are common and distressing symptoms in patients with end-stage chronic kidney disease (ESRD) [12]. About two-fifths of patients with ESRD experience moderate-to-severe pruritus, leading to depression, poor life quality, and increased mortality [13]. Several theories on the development of uremic pruritus have been proposed, including abnormal calcium homeostasis, imbalanced opiate receptors, neuropathic dysregulations, and enhanced systemic inflammation. Since there is no single target in the pathogenesis of uremic pruritus, general preventive measures are important to prevent itch, including aggressive skin hydration, patient education, and wound care. Other treatment modalities have been tried with variable success, including dialysis adequacy, flow rate, and medium selection [14]. Itch managements such as gabapentin, ultraviolet radiation B (UVB) phototherapy, acupuncture, topical steroids, and opioid-receptor modulators may also be effective [15].

### 2.3. Pruritus as A Presentation of Occult Diseases

It is noteworthy that patients with chronic and intractable itch without attributable causes should be evaluated for occult systemic diseases such as thyroid disease, polycythemia vera, Hodgkin disease, and human immunodeficiency virus (HIV) infection [16]. A thorough history-taking and a complete physical examination are important for the evaluation of pruritus [17]. In the absence of skin lesions, additional evaluation should include complete blood counts, liver function tests, serum creatinine tests, blood urea nitrogen levels, measurement of thyroid stimulating hormone, and chest X-rays.

### 2.4. Cholestatic Pruritus

A significant proportion of patients with chronic cholestatic diseases, including biliary cirrhosis and sclerosing cholangitis experience pruritis [18]. Unfortunately, the pathogenesis of pruritus of cholestasis remains to be determined. Candidate pruritogens might include histamine, serotonin, bile salts, endogenous opioids, lysophosphatidic acid (LPA), and progesterone metabolites, however, causative evidences are limited [19,20]. In fact, these molecules may be itch-modifying rather than itch-causing. Due to the lack of strong evidence-based studies and the largely unknown mechanisms of cholestatic pruritus, its treatment options remain very limited. Current consensus treatment plans include sequential use of anion-exchange resins cholestyramine (4–16 g/day), μ-opioid inhibitor naltrexone (50 mg/day), pregnane X receptor-agonist and enzyme inducer rifampicin (300–600 mg/day), and selective serotonin reuptake inhibitors (SSRIs) such as sertraline (100 mg/day) [21]. However, a substantial number of patients are refractory to these drugs and require further interventions including UVB phototherapy, plasma separation, anion absorption, extracorporeal albumin dialysis, or even nasobiliary drainage [21].

## 3. Primarily Inflammatory Diseases

While some inflammatory skin diseases such as lupus erythematosus, cellulitis, and pityriasis lichenoides may or may not cause itch, others such as psoriasis and atopic dermatitis (AD) cause moderate-to-severe itch in a majority of the patients. It is important to find the key mediators that cause such differences. Since AD is common and inevitably causes itch, we review the pathophysiology of this prototypic primarily inflammatory itchy skin disease.

### AD as the Prototypic Inflammatory Model to Cause Pruritus

AD is the prototypic inflammatory skin disease that is always accompanied by intensive itch [22]. The pathogenesis of AD is multifactorial and includes interactions between genetic components, microbiomes, and environmental influences. In addition to the immune responses, the impaired skin barrier in AD facilitates allergen entry and enhances water loss from inside, thus forming a vicious cycle that augments the allergic immune responses [23]. The activation of nerve endings in AD skin by pruritogens, including immune mediators, neuropeptides, and neurotransmitters, results in itch.

## 4. Targeting Itch through Immunological Mechanisms in AD

Physicians have known for many decades that antihistamines barely alleviate itch in patients with AD in the acute phase. However, systemic steroids that block the inflammatory upstream have shown effective in controlling the acute flare and itch of AD. In the subacute phase, the inflammatory itch is controlled by targeting calcineurin, important in T cell activation, by cyclosporin A and topical tacrolimus [24]. To further control the inflammation-related itch in AD, many monoclonal biologics are being or have been developed (Table 1). Of note, it is possible that these cytokine-targeting treatments may alleviate itch not only by direct antipruritic effects but also indirect anti-inflammatory effects.

### 4.1. IL-2

IL-2 is an autocrine cytokine that induces T cell activation. IL-2 may be a cause of itch, as systemic treatment of metastatic melanoma with IL-2 induces severe itch. Cyclosporine, through inhibition of calcineurin activation, inhibits T cell activation mediated by the IL-2 autocrine pathway and therefore reduces inflammation and pruritus in AD [27].

### 4.2. IL-4 and IL-13

IL-4 and IL-13 are two important Th2 cytokines in AD. Their receptors share a common subunit. In mice, transgenic overexpression of IL-4 or IL-13 results in a severe itching, atopic-like dermatitis phenotype [28]. Recently, a mouse study showed that IL-13 mediates the development of pruritus via TRPA1 activation [29]. In skin of human AD, the expression of IL-13 receptor α1 is increased [30]. In blood from patients with AD, the level of IL-13 is increased and correlated with disease severity [31]. A recent clinical trial showed that dupilumab, the monoclonal antibody against IL-4Rα, at 300 mg subcutaneous injection every week for 12 weeks, achieved more than 50% reduction of itch perception in AD and clearly noticeable improvement in disease activity [32]. Lebrikizumab, a monoclonal antibody against IL-13 [33], has been tested in patients with moderate-to-severe AD as a topical steroid treatment in a phase II trial. The results were announced in the recent 2016 European Academy of Dermatology and Venereology (EADV) meeting, showing preferential percentages of eczema area and severity index (EASI)50 in the treatment group versus placebo group (82.4% vs. 62.3%) (clinical trial#NCT02340234).

### 4.3. IL-5

In AD, there is usually blood and tissue eosinophilia. One of the most important cytokines in eosinophil activation is IL-5. A randomized, short-term treatment of patients with AD using meplizumab, a humanized anti-IL-5, showed a reduction in eosinophils. However, treatment outcomes were similar between the treatment and placebo groups [34].

### 4.4. IL-31

In mice, transgenic overexpression of IL-31 in lymphocytes results in severe pruritic atopic-like dermatitis [35]. IL-31, which is expressed preferentially in Th2 cells, activates a heterodimeric receptor formed by IL-31 receptor A (IL-31RA) and oncostatin M receptor (OSMR) in keratinocytes and free nerve endings [36]. The blood level of IL-31 is increased in many pruritic skin diseases including AD, cutaneous T cell lymphoma, uremic pruritus, chronic urticaria, and prurigo nodularis [37]. Furthermore, blood IL-31 level is correlated to disease severity in patients with AD [37]. In skin, expressions of IL-31RA and IL-31 are increased in AD [38]. In line with this, we have demonstrated that IL-31 induces STIM1 activation, followed by STAT3 phosphorylation and β-endorphin release in keratinocytes [39] in peripheral skin. Regarding the central mechanisms of itch, interestingly, dorsal root ganglion neurons coexpress TRPV1 and IL-31R [40]. Similar to the action of TSLP (see Section 4.6), the IL-31-induced itch requires TRPV1 and TRPA1 [40]. Notably, IL-31 induces a late onset of pruritus by hours, suggesting that the itch induction by IL-31 may occur through an indirect mechanism rather than through cutaneous receptor activation [41]. This compelling evidence renders the action to develop a targeted biologic against IL-31 in the itch treatment. A phase I clinical trial is being conducted to test the effect of anti-IL-31 antibody (NCT01614756) [42]. Another phase II trial aims to test multiple doses in 250 patients with AD with pending results (NCT01986933).

### 4.5. IL-17

The involvement of Th17 and its associated cytokines, IL-17 and IL-22, in AD are less known than that of Th2 and its associated cytokines (i.e., IL-4 and IL-13). IL-17A was shown to establish Th2 responses in two AD mouse models [43]. In patients, IL-17 was shown to preferentially associate with acute skin lesions [44] of AD and circulating blood [45]. Ustekinumab, a biologic targeting IL12/23, was tested recently in a phase II study with 33 cases of AD, which showed a superior response in the ustekinumab group versus control group (SCORAD50 = 31% and 16%, respectively, at 16 weeks) [25].

### 4.6. Thymic Stromal Lymphopoietin

Thymic stromal lymphopoietin (TSLP) is an IL-7-like cytokine that triggers dendritic cell-mediated T helper (Th) 2 inflammatory responses [46]. Blood TSLP level is increased in patients with AD [47]. The production of TSLP in keratinocytes may play an important role in the atopic march from AD to the development of asthma [48]. An elegant study showed that the ORAI1/nuclear factor of activated T cell (NFAT) calcium-signaling pathway is an essential regulator of TSLP release from keratinocytes and that TSLP then acts directly on TRPA1-positive sensory neurons to trigger robust itch behaviors in mice [49]. Consistent with this, we have shown that the *ORAI1* genetic polymorphism confers differential susceptibility of AD in Japanese and in Taiwanese AD patients [50]. A phase I study with systemic injection of AMG157, a human monoclonal antibody against TSLP, in patients with AD was finished 4 years ago with pending results (NCT00757042). MK8226, an inhibitor for TSLP receptor, has also been developed and used in a phase I study with patients with AD; however, no results have been released (NCT01096160).

### 4.7. Immunoglobulin E (IgE)

IgE level is elevated in the blood from patients with allergic diseases such as AD, asthma, and allergic rhinitis. Omalizumab is a humanized monoclonal antibody against circulating IgE. It prevents the interaction of IgE and its high affinity receptor (FceRI) on mast cells, basophils, and dendritic cells, altering the antigen presentation process. Omalizumab is approved for the treatment of chronic idiopathic urticarial [51]. Around 80% of patients with AD have elevated blood IgE, thus efforts have been made to test whether blocking IgE can also treat AD. However, the clinical response for IgE-blockage in AD is controversial. Notably, the clinical response is not related to the level of IgE [52]. A new anti-IgE treatment with higher affinity than omalizumab, QGE031 (ligelizumab), has been tested in patients with AD; however, the results are not disclosed yet (NCT01552629).

### 4.8. Protease-Activated Receptors (PAR)

Protease-activated receptors (PARs) belong to the G protein-coupled receptors family and are activated by synthetic peptides that match the sequence of the tethered ligands at the receptor’s N-terminus. The activation of PAR2 and PAR4 has been reported to mediate nonhistamine itch. Both PAR2 and PAR4 are found in keratinocytes and in doral root ganglions (DRG). Expression profiles for both tryptase and PAR2 were significantly increased in lesional skin of AD patients [40]. In addition, PAR-2 was markedly enhanced on primary afferent nerve fibers in skin biopsies of AD patients. Topical treatment with E6005, a phosphodiesterase 4 inhibitor that inhibits PAR2 peptide agonist SLIGRL-NH2, reduced itch suggesting that PAR2-associated itch might be related to lower levels of cAMP and subsequent production of LTB4 [53].

### 4.9. Transient Receptor Potential (TRP) Channels

TRP channels are expressed in several types of skin residential cells, including keratinocytes, melanocytes and nerves. They not only contribute to sensory processing but also to other physiological functions [54]. Several classes of TRP proteins may be involved in itch perception, including TRPV1, TRPA1 and TRPM8 [55].

TRPV1 expression is increased in itching skin lesions and its activation promotes itch by secreting soluble factors [56]. TRPV1 is critical in histamine-dependent itch. Amagai et al. reported that stimulation against TRPV1 reduced scratching behavior in conventional NC/Tnd mice with AD. It suggests that TRPV1 regulates AD itch [57]. However, a recent clinical trial using topical TRPV1 inhibitor (SB705498) failed to relieve itch induced by either histamine or cowage [58].

TRPA1 contributes to the transduction of histamine-independent itch [59]. TRPA1 expression is present in sensory nerves, mast cells, and keratinocytes [60,61]. In AD induced by IL-13, TRPA1 expression is enhanced and contributes to pruritus [61]. TRPA1 is a downstream molecule of MrgprA3 for itch signaling but its pathophysiological effects in itch of AD are complicated in different aspects. On the one hand, it mediates itch signals, but on the other hand, its activation promotes barrier recovery [62]. In IL-31-mediated itch, both TRPV1 and TRPA1 are required [40].

TRPM8 is expressed in both A-δ and C fibers [63,64]. Administration of substance P attenuates the scratching activity induced by menthol, a TRPM8 agonist [55]. In a subset of patients, itch was transmitted through A-Δ fibers but not the common unmyelinated C fibers [65]. This might explain the diversity of itch that patients experience [65].

## 5. Treatment Strategy for Noninflammatory Itch through Neurotransmitters and Neuropeptides in AD and Other Itching Skin Diseases

For these primarily noninflammatory diseases, treatments directed against neuropeptides, neurotransmitters, and other neurological mediators are reasonable methods to control itch (Table 2).

### 5.1. Histamine H1 and H4 Receptor

Histamine, which sources mostly from dermal mast cells, is the major factor in inducing itch in urticaria, insect bites and mastocytosis [73]. Histamine activates H1 receptors on C fibers that, in turn, induce expression of phospholipase Cβ3 [74]. Prior studies showed that the H1-receptor (H1R) is expressed in dorsal root ganglions and that inhibitors of H1R can completely suppress histamine-induced itch in human skin [73]. Thus, the H1 receptor is implicated as an important mediator of histamine-induced itch reactions [75]. In addition to its direct histamine antagonism to alleviate itch, H1R inhibitors were also found to reduce levels of IL-31 in skin lesions of chronic dermatitis in NC/Nga mice and of AD. Other mechanisms of H1 antihistamines to reduce itch include reducing H1 receptor activity, decreasing bradykinin production, and downregulating NF-κB, a proinflammatory transcriptional factor [73]. Olopatadine, a non-sedating H1 antihistamine, decreases nocturnal scratching in AD without affecting sleep quality, suggesting that central sedative effects of antihistamines may not be necessary to alleviate itch in AD and the clinical choice of first generation antihistamines for AD should not depend solely on their sedative effects [76].

H4 receptors have been found to be expressed mainly on mast cells, dendritic cells and eosinophils, all of which are important in the pathophysiology of AD [77]. In H4R-deficient mice, histamine-or H4R-ligand-induced itch response is attenuated [78]. This evidence indicates that the H4 receptor may be important in itch perception in AD. A combination of H1R and H4R antagonists also reduced itch and inflammation, achieving anti-inflammatory effects similar to prednisolone. In a phase II trial, JNJ39758979, a compound that inhibits H4R, has been shown to be effective in treating pruritus in Japanese patients with AD [79].

### 5.2. Acetylcholine

In AD, itch is usually accentuated by perspiration. Postganglionic sweating responses of autonomic cholinergic fibers may be closely associated with the itch perception of sensory unmyelinated C fibers. In mice, activation of the muscarinic M3 receptor mediates pruritus [80]. Both histamine-sensitive and -insensitive C fibers may be activated by acetylcholine. In some patients with intractable pruritus, oral doxepin, an anticholinergic drug, could be effective through central regulatory mechanisms independent of antihistamine actions.

### 5.3. Antidepressants

Tricyclic antidepressants, including amitriptyline, trimipramine, and nortriptyline, are found to be effective against itch. However, these agents have not been studied for itch management in randomized controlled trials (RCT). Doxepin is also a tricyclic antidepressant with antihistamine ability and a small clinical trial demonstrated the effect of doxepin in reducing uremic pruritus [81]. Doxepin may also be useful in HIV-related pruritus as well as itch in chronic urticarial [82]. Note, tricyclic antidepressants should not be combined with monoamine oxidase inhibitors and should be used with caution in elderly patients with glaucoma or benign prostate hypertrophy.

### 5.4. Selective Serotonin Reuptake Inhibitors (SSRI) and Serotonin-Norepinephrine Reuptake Inhibitors (SNRI)

In humans, peripheral injection of serotonin only results in a mild itch by causing histamine release from mast cells [83]. However, treatment with a 5HT3-receptor antagonist improves itch induced by opioids, suggesting a central action for serotonin in itch induction.

Treatment of chronic pruritus with SSRIs, including paroxetine and fluvoxamine, was reported to be effective in a small open-labelled study (*n* = 72) [84]. Sertraline has been shown to be effective in treating itch in primary biliary cirrhosis [85] and in uremic pruritus [86] in small studies. Additionally, the adverse cardiovascular effects are generally lower in SSRI compared to tricyclic antidepressants, although nausea, dizziness, insomnia, and sexual dysfunction may occur. Mirtazapine, another antidepressant, might be useful for itch relief in patients with advanced cancers, ESRD, cholestasis, and nocturnal itch [87]. However, there is currently no RCT for mirtazapine.

### 5.5. γ-Aminobutyric Acid (GABA) Analogs

Gabapentin and pregabalin are structure analogs of the neurotransmitter γ-aminobutyric acid (GABA). Drug onset is earlier for pregabalin. Two RCTs demonstrated that gabapentin at 300–400 mg per day is effective and safe for treating uremic pruritus [88,89]. However, the same could not be demonstrated for cholestatic pruritus [90]. Gabapentin has been tested against itch in several other diseases and at higher doses appeared beneficial in several of these diseases, although the case numbers were low or uncontrolled. Similarly, pregabalin has been tested in 2 RCTs, showing promising results in reducing the uremic pruritus [71,91].

### 5.6. Opioid Receptors

Morphine, a strong pain killer and μ-opioid receptor agonist, induces itch in a substantial proportion of patients. Intraspinal-morphine-induced itch can be inhibited through μ-opioid receptor inhibition [92], indicating that central opioid regulation may contribute to the itch to a large extent. On the other hand, as peripheral histamine release by morphine is independent of μ-opioid receptors and therapeutic doses of opioids are inadequate to induce mast cell degranulation [93], peripheral mechanisms of opioid-induced itch may play a lesser role than central mechanisms. The balance between different subsets of opioid receptors (μ-opioid receptor and κ-opioid receptor) is likely to induce morphine-induced itch. μ-opioid receptor antagonists, such as naloxone and naltrexone, have been shown to reduce intractable itch in patients with uremic pruritus and cholestasis, while κ-opioid receptor agonists, such as nalfurafine, may be effective in reducing itch in patients with ESRD undergoing hemodialysis.

We previously showed that blood level of β-endorphin is associated with itch intensity in patients with AD [94]. The μ-opioid receptor isoform, MOR1D, heterodimerizes with gastrin-releasing peptide receptor (GRPR) co-expressed in itch-signaling spinal neurons [4]. The κ-agonist TRK-820 (nalfurafine) has been shown to inhibit pruritogen-induced scratching [95], suggesting a putative role of the κ-opioid receptor in the regulation of itch. TRK-820 has been proven effective in relieving intractable itch in patients on renal dialysis [96].

## 6. Conclusions

Recent understanding of the pathophysiology of itch helps to develop targeted therapy for itch in AD. Targeted therapy against IL-4, IL-13, TSLP, IgE, IL-31, etc. has been developed and undergone clinical trials. Promising results have been observed in some of them. With the recent development of targeted therapy for inflammatory itch in AD, it may be possible to treat intractable itch in these patients in the near future. On the other hand, several other itchy skin diseases, such as uremic pruritus and cholestatic pruritus, remain treatment challenges, partly due to unknown major targets. Phototherapy and pharmaceutical interventions, such as tricyclic antidepressants, GABA inhibitors, SNRIs, and SSRIs, may be helpful in some of them. Further multidisciplinary research towards the understanding of the pathophysiology of itch in these diseases is warranted to develop appropriate treatment against the troublesome itch.

## Figures and Tables

**Table 1:** Potential targeted biologics against inflammatory pruritus in atopic dermatitis (AD).

The Targets Based on the Mechanism of AD		
Th2 Axis		
IL-4Ra	Duplimumab	Phase II, *n* = 197, EASI score reduction at 16 weeks: 72% vs. 38%
IL-13	Lebrikizumab	Phase II, *n* > 200, EASI50: 82.4% vs. 62.3%
IL-31	BMS-981164	Phase I, NCT01614756
IL-31RA	CIM331	Phase II, *n* = 264, pruritis score 50% reduction: 40% vs. 20%
Th17 axis		
IL-17	Secukinumab	Phase II, ongoing (NCT02594098)
IL12/23	Ustekinumab	EASI50 at 16 weeks *n* = 3 [25] Phase 2: SCORAD50 at 16 weeks: 31% vs. 16% *n* = 33 [26]
IL-22	ILV-094	Phase II, ongoing (NCT01941537)
Epidermis		
TSLP	AMG157	Phase I, RCDB, *n* = 157 (NCT00757042)
TSLPR	MK8226	Phase I, completed, *n* = 40 (NCT01096160)

IL, Interleukin; EASI, Eczema Area and Severity Index; TSLP, Thymic stromal lymphopoietin; TSLPR, Thymic stromal lymphopoietin receptor; SCORAD, Severity Scoring of Atopic dermatitis Index.

**Table 2:** Perspectives in the treatment of pruritus in AD and other itching skin diseases.

Candidate Mediators of Itch	Drugs
Histamines	H1 antihistamine
	H4 antihistamine
Leukotrienes	Zafirlukast and Aileuton [66]
Prostaglandins	NSAID for opioid-induced pruritus [67]
Acetylcholine	Botulinum toxin type A for histamine-induced itch [68] Doxepin via anticholinergic action
Neurokinin Receptor 1	Aprepitant for refractory pruritus [69]
GABA	GABA inhibitors: Gabapentin, Pregabalin
Serotonin	SSRI: Paroxetine, Fluoxetine Serotonin agonist: Mirtazipine for uremic pruritus [70] 5HT-3 antagonist: Ondansetron for uremic pruritus [71]
Opioids	Naloxone and Naltrexone Nalfurafine and Butarphanol for uremic pruritus [72]
TRP channels	TRPV1: Capsaicin TRPM8: Menthol

NSAID, Non-Steroid Anti-Inflammatory Drug; GABA, γ-Aminobutyric acid; SSRI, Selective serotonin reuptake inhibitor; TRP, Transient receptor potential; TRPV, vanilloid receptor–related transient receptor potential.
